# A national study on the resilience of community pharmacists in Lebanon: a cross-sectional survey

**DOI:** 10.1186/s40545-022-00406-2

**Published:** 2022-01-28

**Authors:** Mohamad Alameddine, Karen Bou-Karroum, Mohamad Ali Hijazi

**Affiliations:** 1 College of Health Sciences, University of Sharjah, Sharjah, United Arab Emirates; 2 College of Medicine, Mohammed Bin Rashid University of Medicine and Health Sciences, Dubai Health Care City, Dubai, United Arab Emirates; 3 Department of Health Management and Policy, Faculty of Health Sciences, American University of Beirut, Riad El Solh, Beirut, Lebanon; 4 Department of Pharmaceutical Sciences, Faculty of Pharmacy, Beirut Arab University, Beirut, Lebanon

**Keywords:** Resilience, Retention, Community pharmacists, Lebanon

## Abstract

**Background:**

Community pharmacists are among the most accessible healthcare professionals and are likely to experience the full brunt of public health crises. In Lebanon, the COVID-19 pandemic, added to a severe economic meltdown, have significantly disrupted an already suffering profession.

**Methods:**

The objective of this study was to determine the level of resilience and its relationship to burnout, job satisfaction, intention to quit, and changes in practice. The study utilized a cross-sectional design to survey community pharmacists using an online questionnaire that included the Connor-Davidson Resilience Scale and the Copenhagen Burnout Inventory. All community pharmacists were invited to participate. Multiple logistic regression identified variables significantly associated with the resilience of pharmacists.

**Results:**

A total of 459 community pharmacists completed the questionnaire. Respondents had a relatively low resilience level (68.0 ± 13.37). They also had higher scores on the client-related burnout (58.06 ± 17.46), followed by the personal burnout (56.51 ± 16.68) and the work-related burnout (55.75 ± 13.82). In this sample, 52.3% of pharmacists indicated that they are dissatisfied with their job and 41.1% indicated an intention to quit in the coming year. According to multivariate analysis, marital status (*ß* = 0.38; 95% CI 0.16–0.91; *p* = 0.03), intention to quit (*ß* = 0.384; 95% CI 0.149–0.987; *p* = 0.047), workload (*ß* = 0.275; 95% CI 0.096–0.783; *p* = 0.016), perception of safety (*ß* = 0.267; 95% CI 0.078–0.909; *p* = 0.035), and personal burnout (*ß* = 0.321; 95% CI 0.152–0.677; *p* = 0.003) were independent influencing factors for resilience.

**Conclusions:**

Multiple challenges and crises have culminated to the low job satisfaction, high burnout, and high the intention to quit of community pharmacists. This seriously destabilized the labor market of pharmacists which could negatively affect public safety. Effective interventions are essential to enhance the well-being and job satisfaction of pharmacists during public health crisis.

## Background

Community pharmacists are members of the healthcare team that have a significant role to play in dealing with public health crises including pandemics [1]. They have a unique credible role in the global response to pandemics across four key phases: prevention, preparedness, response, and recovery [1]. During public health crises, community pharmacists are among the most accessible healthcare workers and are likely to experience the full brunt of the crisis [1, 2]. At times when physicians are overworked and emergency rooms are overwhelmed, community pharmacists are often the first point of contact with the health system for many patients who need reliable health information and advice [2]. The impact of the corona virus disease 2019 (COVID-19) pandemic has been profound on all health professions, pharmacists are no exception [1]. Community pharmacists deliver medications to patients, contribute to COVID-19 screening, help with the administration of vaccines, and elucidate misconceptions about COVID-19 treatments [3]. Strict lockdowns result in an even greater dependence on pharmacists, since pharmacies are one of the few places that remain open for public service.

Literature emphasized the importance of including community pharmacists in the pandemic planning protocols as they could play a vital role in society-wide pandemic preparedness [2]. Recently, the “Fédération Internationale Pharmaceutique” (FIP) published guidelines for the pharmacy workforce highlighting core responsibilities associated with the provision of public health services, patient education, and vaccine administration during the COVID-19 pandemic [4].

Globally, pharmacists and other healthcare workers who are at the frontline against COVID-19, reported experiencing the negative psychological effects of the pandemic, such as severe stress, fear, anxiety, and burnout [5]. The increased burden on pharmacists’ roles jeopardized their psychological and mental well-being [5]. In fact, unexpected surges in patients seeking medication counseling/health advice, increased risk at work, and increased in drug shortages, are among the multiple challenges reported by pharmacists during the pandemic [6]. Such challenges were exacerbated by the shortage of personal protective equipment, lack of information related to the virus, and lack of clear policies to guide the daily work of hard working community pharmacists [6].

Under such difficult working conditions, community pharmacists are at higher risk of experiencing high burnout and post-traumatic stress symptoms [7]. Occupational burnout has been associated with reduced work productivity, lower job satisfaction, increased turnover, and poorer health outcomes [8, 9]. If unaddressed, burnout may have more serious long-term consequences on the labor market of pharmacists after the pandemic is over.

Literature further highlighted the importance of resiliency and adaptability of frontline practitioners to effectively respond to the rapidly changing contexts of the pandemic [2]. Resilience has been defined as the ability to adapt to substantial sources of stress, using resources within the individual or environment to cope with adversity [10]. Several studies revealed that resilience acts as a protective factor against burnout and other psychological outcomes [11–13]. The association between resilience, burnout, job satisfaction and turnover intention has been extensively examined in previous studies among healthcare professionals, especially among nurses [11–13]. However, there is dearth of research on resilience among community pharmacists.

### The local context

Lebanon, a small country in the Eastern Mediterranean Region, has endured several wars, civil unrests, and conflicts over the last few decades. However, the recent crisis, which started in October 2019, is considered the worst in the history of the country. This is because the country has been facing an ongoing economic collapse, civil unrest, political instabilities, an infectious disease outbreak, and more recently, a massive explosion that ripped through the city’s port [14]. Even before the country went into the abovementioned crises, Lebanese pharmacists were quite dissatisfied and have been facing multiple challenges related to the organization of the profession and the protection of their professional status [15, 16]. Recent studies reported the challenging work context of community pharmacists with issues including unethical competition, policy violations (e.g., employing non-pharmacists to dispense drugs), and practices that jeopardize public safety (e.g., dispensing medications without a prescription) [15, 17]. More recently, the financial crisis forced more than 700 pharmacies to permanently shut down as pharmacists have been struggling with a dangerous shortage in pharmaceuticals, drugs, and medical supplies, coupled with poor profitability [18]. During such dire times, supportive strategies and policies are imperative to strengthen community pharmacists and enhance their resiliency.

Given the magnitude of the pandemic and its adverse consequences on the pharmacy profession, it is important to assess the psychological impact of the pandemic on community pharmacists. The objective of this study was to investigate the resilience level of the pharmacy workforce and its relationship to perceived burnout, job satisfaction, turnover intention, and changes in practice (e.g., workload, income, and perception of safety).

## Methods

### Study design and sampling

A cross-sectional design was utilized to survey community pharmacists in Lebanon. According to the registration database of the Order of Pharmacy in Lebanon (OPL), 4,112 community pharmacists were actively practicing at Lebanese pharmacies at the time of the study. Sample size calculations showed that to estimate a prevalence of resilience of 50%, with a 95% confidence interval (CI) and a margin of error of 5%, a minimum sample of 352 pharmacists were needed for this study [19]. The 50% prevalence of resilience was assumed due to the lack of previous studies that have measured the degree of resilience of pharmacists in Lebanon and because this will render the highest sample size in this study. Pharmacists were included in this study if they are licensed to practice by Ministry of Public Health (MoPH), registered with the OPL, working in a pharmacy whether as pharmacy owner or as an employee, and conversant in either English or Arabic languages.

### Survey instruments

A five-segment questionnaire was utilized for data collection. The first segment consisted of socio-demographic characteristics, such as age, gender, marital status, level of education, and employment status. In the second segment, pharmacists were asked to rate their job satisfaction on a 5-point Likert scale. They were also asked about their intention to quit their current workplace in the coming year; followed by a question probing their plans if they intend to quit. The third segment included self-reported questions related to pharmacists’ practice during the COVID-19 pandemic (e.g., workload, perception of risk, salary, and safety). The fourth segment entailed a 25-items resilience scale developed by Connor and Davidson (CD-RISC) to assess resilience among respondents. Note that the scale was used with the permission of the authors [20]. Pharmacists were requested to answer using a 5-point Likert scale with 0 = not true at all and 4 = true nearly all the time. The scale showed good reliability and validity scores, with Cronbach alpha value calculated as 0.89 [21].

The last segment of the questionnaire included the Copenhagen Burnout Inventory (CBI) to measure professional burnout among respondents. The scale consists of 19-items with 3 subscales: personal burnout (6 items), work-related burnout (7 items), and client-related burnout (6 items). Personal burnout measures burnout from an individual perspective related to n individual’s degree of physical and psychological exhaustion and fatigue, whereas work-related burnout and client-related burnout measure burnout as related to the individual’s work and that related to the relationship with clients, respectively [22]. Items are scored on a 5-point Likert scale from always to never. The Cronbach alphas for the Copenhagen Burnout Inventory subscales were calculated as 0.85–0.87 [23], indicating good internal consistency. The scales were translated to Arabic language and back-translated to English by experts. A pilot test was conducted with 10 pharmacists to validate the questionnaire, no significant changes were introduced to the questionnaire following pilot testing. The online questionnaire was developed using Lime Survey software [24].

### Data collection

The study utilized an online survey tool to collect data between December 2020 and January 2021. A deidentified list of contact numbers of community pharmacists was obtained from the OPL. An invitation letter was sent along with a consent form and the online questionnaire to all community pharmacists via short message service (SMS). Only consenting pharmacists were able to complete the questions which took an average of 10 min to complete and was available in both English and Arabic.

### Data analysis

The collected data were analyzed using the Statistical Package for Social Sciences software (IBM SPSSv.24). Descriptive statistics including frequencies, mean, and standard deviation were generated to describe the characteristics of the study population, such as the pharmacists’ burnout levels, degree of resilience, and intention to quit. Simple logistic regression was carried out to determine the independent variables that were significantly associated with resilience. To adjust for confounders, the variables that were significantly associated with resilience in the simple logistic regression were entered into a multiple logistic regression model. Quartiles were used to analyze the CD-RISC resilience scale [25]. Pharmacists were grouped in three groups: low resilience (participants falling in the lowest quartile), moderate resilience (participants belonging to the second and third quartiles), and high resilience (participants falling in the highest quartile) [25]. The total score for each of the three CBI burnout subscales is the mean value obtained from the individual parts. For the sake of statistical analysis, we have chosen CBI scores of 50 or below to be categorized as low burnout and scores higher than 50 to categorize as high burnout [22, 26]. Higher scores for each subscale indicated more burnout. All analysis was carried at 0.05 significance level.

### Ethical considerations

Ethical approval was obtained from the Institutional Review Board (IRB) (2020-H-0067-P-R-0406). Participants were asked to electronically sign the consent form before filling the questionnaire. Participation was entirely voluntary and there were no risks or harms resulting from completing the questionnaire. All ethical issues of anonymity and confidentiality of participants were assured in this study.

## Results

A total of 459 pharmacists completed the questionnaire. The demographic distribution of respondents and their professional characteristics are presented in Table 1. Among the participants, 235 were females (60%), 223 were below 45 years (69.5%), and 341 were ever married (74.8%) (married at least once in their lives). As for the educational level, 69% of respondents graduated from universities inside Lebanon. While close to half of the respondents reported holding a bachelor’s degree, 22.7, 25.5 and 4.1%, reported holding a master’s degree, a Pharm D and a PhD, respectively. A total of 62% of respondents reported more than 10 years of experience. The majority of respondents were pharmacy owners (63.5%), while the remaining were either working as full time (29.1%) or part time (7.4%). These results are relatively similar to the national distribution of community pharmacists among the various governorates, suggesting that this sample is representative of the general pharmacy workforce [17].

**Table 1:** Demographic and professional characteristics of the study population

Variable	Characteristics	Frequency (*N* = 459)	Valid Percent
Gender	Female	235	60.1
	Male	156	39.9
Age	20–29 years	96	20.9
	30–45 years	223	48.6
	46–55 years	92	20.0
	More than 55 years	48	10.5
Marital status	Ever married	341	74.8
	Never married	115	25.2
Highest education	BSc Pharmacy	218	47.6
	Pharm D	117	25.5
	Masters	104	22.7
	PhD	19	4.1
Years of experience	Less than 5 years	69	15.2
	5–10 years	104	22.9
	More than 10 years	282	62.0
Governorate	Beirut	89	19.4
	Mount Lebanon	138	30.1
	North Lebanon	91	19.8
	South (including Nabatieh)	87	19.0
	Beqaa	54	11.8
Employment status	Pharmacy owner	290	63.5
	Full time	133	29.1
	Part time	34	7.4
What is the location of the university you graduated from?	Inside Lebanon	318	69.3
	Outside Lebanon	141	30.7

As displayed in Table 2, slightly more than half of the responding pharmacists (52.3%) were dissatisfied with their job, 28% were satisfied, and 19.7% were neutral. Table 2 also reveals that a sizable proportion of responding pharmacists (41.1%) indicated that they would likely/very likely quit their current workplace in the next 12 months. Among those planning to quit, about two thirds reported that they would like to migrate outside Lebanon (66.1%), shift to work in another pharmaceutical sector inside Lebanon (14.2%), stop working (13.4%), or work in a non-health organization (6.3%).

**Table 2:** Analysis of job satisfaction and intention to quit

	Frequency (*N* = 459)	Valid percent
Job satisfaction		
Satisfied	115	28.0
Neutral	81	19.7
Dissatisfied	215	52.3
Missing	48	
Intention to quit over the next 12 months		
Very unlikely	95	23.8
Unlikely	141	35.3
Likely	105	26.3
Very likely	59	14.8
Missing	59	
If you are intending to quit, would you like to:		
Migrate from Lebanon	261	66.1
Stop working	53	13.4
Work in a non-health organization	25	6.3
Work in the profession of pharmacy at another institution or sector in Lebanon	56	14.2
Missing	64	

The results of the questions assessing the impact of COVID-19 pandemic on the community pharmacists’ practice are displayed in Table 3. An overwhelming majority of pharmacists (89.6%) believed that they are at risk, of which 59.3% perceived the risk to be moderate and 30.3% perceived the risk to be high. Similarly, 83.3% of responding pharmacists reported that they felt less safe during the COVID-19 pandemic, while only a small proportion reported that they feel safer (9.9%) or reported no change in safety (6.8%). Regarding workload, 70.8% of respondents reported an increase in their workload because of the pandemic, while the remaining reported no change in their workload (17.2%) or a decreased workload (12%).

**Table 3:** Analysis of COVID-19-related factors in the study population

	Frequency (*N* = 459)	Valid percent
Perception of risk		
No risk	12	3.1
Low risk	28	7.3
Moderate risk	227	59.3
High risk	116	30.3
Impact on income		
Income decreased	145	37.8
Income increased	87	22.7
No change in income	152	39.6
Impact on workload		
Workload increased	271	70.8
No change in workload	66	17.2
Workload decreased	46	12.0
Impact on safety		
Less safe	320	83.3
No change in safety	26	6.8
Safer	38	9.9

Community pharmacists in this sample had a mean CD-RISC resilience score of 68.0 ± 13.37, with total scores ranging from 18 to 100 (Table 4). The resilience levels of pharmacists were differentiated using quartile scores. The average levels of burnout as per the CBI among responding pharmacists were divided into low and high burnout groups. Pharmacists had higher scores on the client-related burnout (58.06 ± 17.46), followed by the personal burnout (56.51 ± 16.68) and the work-related burnout (55.75 ± 13.82). High levels of personal, work-related, and client-related burnout were found among 56.7%, 58.2%, and 57% of pharmacists, respectively.

**Table 4:** Analysis of resilience and burnout levels in the study population

Analysis of the CD-RISC results	
Mean (SD)	68.0 ± 13.37
Minimum	18
Maximum	100

	Frequency (*N* = 459)	Valid percent
First quartile	91	27.3
Second quartile	81	24.3
Third quartile	79	23.7
Fourth quartile	82	24.6
Missing	126	

Analysis of the CBI results			
	Frequency (*N* = 459)	Valid percent	Mean (SD)
Personal burnout			56.51 ± 16.68
Low	120	43.3	
High	157	56.7	
Missing	182		
Work-related burnout			55.75 ± 13.82
Low	84	41.8	
High	117	58.2	
Missing	258		
Client-related burnout			58.06 ± 17.46
Low	102	43	
High	135	57	
Missing	222		

The association between resilience and the sociodemographic and professional characteristics as derived from simple and multiple logistic regression are displayed in Table 5. Simple and multiple logistic regression results indicated that among all sociodemographic characteristics considered in this study, marital status was the sole predictor of resilience. Those who were never married had significantly lower resilience levels (*ß* = 0.38; 95% CI 0.16–0.91; *p* = 0.03). Table 5 also shows the association of psychological and professional variables with resilience. Pharmacists who indicated that upon turnover they are willing to work as a pharmacist in other places had significantly higher resilience scores as compared to pharmacists who preferred to migrate from Lebanon (*ß* = 3.934; 95% CI 1.031–15.006; *p* = 0.045). No significant association was observed between the “very unlikely to quit” and the “very likely to quit” groups. Pharmacists indicating workload reduction had significantly lower resilience levels compared with those who indicated increase in workload (*ß* = 0.275; 95% CI 0.096–0.783; *p* = 0.016). Similarly, pharmacists who experienced no change in safety had significantly lower resilience levels compared to their counterparts who indicated feeling less safe (*ß* = 0.267; 95% CI 0.078–0.909; *p* = 0.035). Personal burnout retained statistically significant association with lower resilience levels (*ß* = 0.321; 95% CI 0.152–0.677; *p* = 0.003). In this sample, work-related and client-related burnout were not significantly associated with resilience.

**Table 5:** Association of sociodemographic and professional characteristics with resilience in the study population as derived from the bivariate analysis

Variable	Simple logistic regression		Multiple logistic regression	
	*ß*; 95% Confidence Interval	*P*-value	*ß*; 95% Confidence interval	*P* value
Gender				
Male	0.809 (0.479–1.367)	0.428		
Constant	2.756	0.000	0.517 (0.251–1.067)	0.074
Age				
20–29 years		0.305		0.993
30–55 years	1.487 (0.837–2.641)	0.176	1.058 (0.382–2.928)	0.914
More than 55	1.863 (0.705–4.928)	0.210	1.025 (0.219–4.799)	0.975
Constant	1.917	0.010		
Marital status				
Never married	0.577 (0.342–0.974)	**0.040**	0.384 (0.162–0.910)	**0.030**
Constant	3.085	0.000		
Intention to quit				
Very unlikely		**0.050**		0.171
Unlikely	0.402 (0.194–0.833)	**0.014**	0.384 (0.149–0.987)	**0.047**
Likely	0.394 (0.183–0.849)	**0.017**	0.560 (0.205–1.529)	0.258
Very likely	0.353 (0.151–0.822)	**0.016**	0.866 (0.254–2.952)	0.818
Constant	5.667	0.000		
Quit where				
Migrate from Lebanon		0.134		0.056
Stop working	1.153 (0.560–2.375)	0.700	0.848 (0.314–2.288)	0.744
Work outside health sector	1.009 (0.372–2.737)	0.987	0.286 (0.064–1.276)	0.101
Work as pharmacist in other places	2.954 (1.197–7.290)	**0.019**	3.934 (1.031–15.006)	**0.045**
Constant	2.313	0.000		
Impact of COVID-19 on workload				
Workload increased		0.054		0.022
No change to workload	2.127 (0.989–4.576)	**0.054**	1.754 (0.654–4.708)	0.265
Workload decreased	0.665 (0.330–1.338)	0.253	0.275 (0.096–0.783)	**0.016**
Constant	2.507	0.000		
Impact of COVID-19 on safety				
Less safe		0.087		0.040
No change in safety	0.607 (0.252–1.459)	0.264	0.267 (0.078–0.909)	**0.035**
Safer	2.730 (0.927–8.038)	**0.068**	3.145 (0.586–16.887)	0.182
Constant	2.564	0.000		
Personal burnout level				
High personal burnout	0.489 (0.282–0.849)	**0.011**	0.321 (0.152–0.677)	**0.003**
Constant	3.480	0.000		

Significant *P*-values (< 0.05) are bolded

## Discussion

To the authors’ best knowledge, this is the first study to report the level of resilience and burnout and the associated factors among community pharmacists in Lebanon. The study reports that the average resilience of community pharmacists was 68.0 ± 13.37. Significant determinants of resilience included marital status, intention to quit, workload, perception of safety, and personal burnout. Study findings revealed that the pharmacy workforce is dissatisfied (52.3%) and destabilized (41% likely or very likely to quit their jobs in the next year).

In this study, 41% of community pharmacists indicated an intention to quit their current job in the next 12 months, and 85.8% of the respondents were planning to leave the Lebanese labor market either to migrate outside the country, seek early retirement, or work in a non-health organization. The turnover intention of Lebanese pharmacists is lower than that in Saudi Arabia, whereby 61.2% of pharmacists reported their intention to leave their current job [27]. Previous studies highlighted several factors that might be attributable to pharmacists’ intention to quit, including: place of practice, workload, income and benefits [27], and organizational identification (feeling that they are valuable to the organization and the public) [28]. A major attrition from the market would result in pharmacists’ shortage, which could in turn destabilize the labor market, impact the sustainability of the pharmacy workforce, and negatively affect patient safety. A high turnover of community pharmacists will only exacerbate the current critical circumstances and further weaken the healthcare system and the pharmacy profession. Consequently, the Lebanese population (especially in peripheral or remote areas) would be at risk of having restricted access to pharmaceutical services.

The satisfaction of community pharmacists in Lebanon (28%) was much lower than that of their counterparts in Pakistan (77% satisfied) [29], and Saudi Arabia (64% satisfied) [27]. However, it is noteworthy that the data collected for those studies was prior to the pandemic. Recent studies from Lebanon have identified significant challenges in the pharmacy practice that affect pharmacists’ job satisfaction and their turnover intention [15]. The high rates of job dissatisfaction among community pharmacists raise concerns related to their quality of life and the associated quality of services delivered. Job satisfaction has a direct impact on the mental and psychological well-being of pharmacists [27]. Likewise, previous studies showed that lower job satisfaction and the presence of work-related stressors are significantly associated with pharmacists’ turnover intention [27]. In addition, pharmacists’ job satisfaction is closely linked to the safety of medication dispensing [30]. Given the vital role of pharmacists during the ongoing pandemic, it is essential to implement evidence-based improvements to the working conditions of pharmacists in Lebanon to enhance their retention and prevent further attrition. Pharmacy stakeholders should heed these study findings and devise and implement a national strategy for the improvement of the working conditions of community pharmacists.

The ongoing economic crisis have further deteriorated the job satisfaction of community pharmacists in Lebanon. This is because the profitability of pharmacists in Lebanon is directly related to the pricing and sales volume of pharmaceutical drugs. While the local currency has lost more than 90% of its value [31], the profit of community pharmacists has not increased, and remained at the official exchange rate of local currency to US dollars at the time when the pharmacy operating expenses have increased several fold [32]. This has led more than 700 pharmacies to shut their doors [18], with many more expected to follow in the absence of swift interventions by concerned stakeholder to stabilize the market. The findings of this study, when viewed in context, are quite worrisome and constitute an urgent call for action by all pharmacy stakeholders in Lebanon, including the MOPH, the OPL and academic institutions. A revision of the current business model for the compensation of community pharmacists is necessary to prevent further attrition of pharmacists and deterioration of the profession.

Our findings also suggested that the COVID-19 pandemic has significantly impacted pharmacists’ satisfaction, but the socioeconomic factors and government instability could be the main influencing factors to their intention to leave. In this sample, only 30% of community pharmacists perceived their risk as high, 62% reported that their incomes were not changed or increased, and 70.8% reported increase in their workload. However, 83.3% of pharmacists reported working in less safe conditions. These findings are consistent with studies investigating the challenges experienced by pharmacists during the pandemic [33]. The additional workload imposed by the pandemic on pharmacists is another risk factor for burnout [33]. Indeed, and consistent with other studies, community pharmacists were afraid of getting the virus or passing it on to their loved ones [34]. As such, extra pressure from the pandemic might have imposed additional burdens on pharmacists and led to difficulties with work–life balance, and consequently, reduced their job satisfaction.

Resilience of community pharmacists was relatively low and was coupled with high burnout rates. Comparison with similar studies was not possible due to the dearth of studies using the same tool to assess resilience among community pharmacists during the pandemic. However, pharmacists in our study had lower resilience levels as compared to frontline nurses working in Lebanese hospitals during the pandemic [35]. The low resilience levels of community pharmacists may be attributable to multiple factors that have cumulated over the years. Evidence suggests that the work environment and work-related policies are more integral to the ability of pharmacists to effectively cope and adapt to difficult situations [6]. Previous studies revealed that the factors potentially contributing to the low resilience of Lebanese pharmacists are poor recognition, lack of regulatory policies, and limited profitability [15]. Community pharmacists around the world have been playing a vital role in keeping the pandemic at bay. Yet, the efforts of Lebanese community pharmacists have been relegated and often overlooked when frontline healthcare workers are heralded.

The aforementioned challenges have thus heightened burnout levels among community pharmacists. Our findings revealed that 56.7%, 58.2%, and 57% of pharmacists have high personal, work-related, and client-related burnout, respectively. A systematic review revealed that burnout among pharmacists ranged from 19 to 37% [36]. There is less data available about the burnout of community pharmacists during the COVID-19 pandemic using the same scale. A recent study using the Maslach Burnout Inventory reported high burnout levels, specifically, 25% for emotional exhaustion, 34.9% for depersonalization, and 3% for personal accomplishment [37]. The phenomenon of high burnout among healthcare workers during stressful events has been reported in several recent studies around the world [38, 39]. A significant proportion of pharmacists reported increased workload, decreased income, and less safe environment. Consistent with other studies [33], this difficult working context might have contributed to the high burnout levels among community pharmacists. A burnt-out pharmacy workforce has a significant negative impact on pharmacists, patients, and the healthcare system. Study findings revealed a negative correlation between personal burnout and resilience levels. Consistent with prior evidence [40, 41], resilience in this study has a protective role against burnout. This calls for swift action from relevant stakeholders to regularly assess pharmacists’ resiliency and to design, implement and evaluate programs and policies that would enhance the resilience of pharmacists in Lebanon.

Findings of the multivariate analysis also showed that marital status, intention to quit, workload, and perception of safety were significantly associated with resilience levels. Pharmacists who were never married showed significantly lower resilience. It is evident that marriage serves as a stabilizing element during difficult times [42]. In addition, this could be attributed to the benefits provided by the OPL to the pharmacists’ families, such as insurance plans, retirement benefits, and other incentives. Interestingly, respondents who wanted to work as pharmacists in other places showed higher resilience levels than those who wanted to migrate. This indicates that those pharmacists are trying to cope with all the challenges as they are devoted to their profession. In this study, decreased workload was associated with lower resilience. This is in contrast with evidence from the literature, whereby higher workload is associated with lower resilience and may be predictive of job outcomes, such as burnout and turnover intention [43]. However, this could be explained by the business model of community pharmacy, whereby pharmacists’ income is directly related to the sales volume of pharmaceutical drugs. As such, higher workload would result in a better income, leading to more satisfaction and higher resilience. Pharmacists who experienced no change in safety had significantly lower resilience levels compared to their counterparts who indicated feeling less safe. This is also in contrast with findings in the literature, whereby feelings of vulnerability of contracting the virus were associated with higher stress and lower resilience levels [44].

## Limitations

The current study has a number of shortcomings that are worth mentioning. First, the cross-sectional nature of the study only supports the establishment of associations but not causality which would require more rigorous methodologies. Second, we did not use random sampling; instead, the questionnaire was sent to all community pharmacists providing an equal opportunity for all to participate. As such, it cannot be ascertained that respondents were not different from non-respondents. Third, although the questionnaire was pilot tested, there remains the possibility that some of the questions may not have been fully understood by the pharmacists.

## Conclusion

Community pharmacists have experienced changes to their practice during the global COVID-19 pandemic. Community pharmacists in Lebanon have relatively low resilience, high burnout levels, and were challenged by increased workload, reduced income, and risk of infection. These factors coupled with the difficult circumstances in the country contributed to their job dissatisfaction and increased their turnover intention. Pharmacy stakeholders have a crucial obligation to review and upscale the business model of community pharmacies and to improve the working conditions of pharmacists. Pharmacy stakeholders should regularly assess the resilience of community pharmacists and implement effective interventions for enhancing their job satisfaction and well-being especially at times of public health crisis.

## Data Availability

The data sets generated and/or analysed during the current study are not publicly available due privacy and ethical restrictions but are available from the corresponding author on reasonable request.
